# Engineering a genetically-encoded SHG chromophore by electrostatic targeting to the membrane

**DOI:** 10.3389/fnmol.2014.00093

**Published:** 2014-11-27

**Authors:** Yuka Jinno, Keiko Shoda, Emiliano Rial-Verde, Rafael Yuste, Atsushi Miyawaki, Hidekazu Tsutsui

**Affiliations:** ^1^Laboratory of Integrative Physiology, Graduate School of Medicine, Osaka UniversitySuita, Japan; ^2^Laboratory for Cell Function Dynamics, Brain Science Institute, RIKENWako, Japan; ^3^Department of Biological Sciences, Neurotechnology Center, Columbia UniversityNew York, NY, USA; ^4^Formation of and Information Processing by Neural Networks, and Control, PRESTO, Japan Science and Technology Agency (JST)Kawaguchi, Japan; ^5^Department of Material Science, Japan Advanced Institute of Science and TechnologyNomi, Japan

**Keywords:** fluorescence protein, mutagenesis, electrostatic surface potential, second harmonic generation

## Abstract

Although second harmonic generation (SHG) microscopy provides unique imaging advantages for voltage imaging and other biological applications, genetically-encoded SHG chromophores remain relatively unexplored. SHG only arises from non-centrosymmetric media, so an anisotropic arrangement of chromophores is essential to provide strong SHG signals. Here, inspired by the mechanism by which K-Ras4B associates with plasma membranes, we sought to achieve asymmetric arrangements of chromophores at the membrane-cytoplasm interface using the fluorescent protein mVenus. After adding a farnesylation motif to the C-terminus of mVenus, nine amino acids composing its β-barrel surface were replaced by lysine, forming an electrostatic patch. This protein (mVe9Knus-CVIM) was efficiently targeted to the plasma membrane in a geometrically defined manner and exhibited SHG in HEK293 cells. In agreement with its design, mVe9Knus-CVIM hyperpolarizability was oriented at a small angle (~7.3°) from the membrane normal. Genetically-encoded SHG chromophores could serve as a molecular platform for imaging membrane potential.

## Introduction

Second harmonic generation (SHG), or frequency-doubling, is a second-order non-linear optical phenomena, in which light with twice the frequency of the incident light is generated upon the interaction of matter and intense light (Shen, 1989). Second-harmonic imaging microscopy (SHIM; Campagnola et al., 2001; Millard et al., 2003a) uses SHG as a contrast mechanism and shares some advantages with two-photon excited fluorescence microscopy, such as 3D optical sectioning, a critical benefits for biological applications. But an important feature of SHIM which is not shared by two-photon microscopy (itself a third-order non-linear optical technique) is that SHG signals arise only from non-centrosymmetric media, such as oriented polymers or interfaces. Also, because SHG is a coherent scattering process, SHIM does not necessarily require exogenous dyes. In fact, endogenous microtubule, myosin and collagen fibers produce significant SHG signals (Campagnola et al., 2002). SHIM from membranes and other interfaces, however, generally requires labeling with exogenous π-conjugated chromophores possessing substantial non-linear hyperpolarizabilities.

Taking advantage of its sensitivity to local centrosymmetry, SHIM has been used to distinguish juxtaposed regions of membrane vesicles from un-adhered region (Moreaux et al., 2000) and to detect the exocytotic wave at fertilization in a sea urchin egg (Millard et al., 2005). Such measurements are not easily achievable with two photon excitation microscopy. In addition, SHG offers remarkable advantages for neuroscience because the second harmonics from membrane-bound dyes can be sensitive to trans-membrane voltage (Campagnola et al., 1999; Millard et al., 2003b, 2004; Nemet et al., 2004). This has enabled measuring membrane potential in small membrane structures such as dendritic spines (Nuriya et al., 2006) and axons (Nuriya and Yasui, 2010). For FM4-64, a diffusible SHG indicator, this voltage sensitivity is due to an electro-optic effect (Jiang et al., 2007).

In spite of such potential advantages of SHIM, the development of genetically-encoded SHG chromophore remains in its infancy. Since GFP-like chromophores exhibit non-linear hyperpolarizability, they have been explored as candidates for SHG (Lewis et al., 1999; Khatchatouriants et al., 2000; Roorda et al., 2004; Asselberghs et al., 2008). In past work, signals at the second-harmonic band have been reported in *C. elegans* expressing GFP tagged with a transmembrane protein (Lewis et al., 1999; Khatchatouriants et al., 2000). But the nature of chromophore arrangement at the membrane interface, the most critical factor in the design of SHG chromophores, remained unclear. Also, this construct was not expressed in mammalian cells. Attempting to generate a defined geometry of GFP chromophores at the membrane interface, Roorda et al. (2004) generated a dually tagged EGFP by incorporating prenylation and palmitoylation signal sequences simultaneously into the EGFP sequence. While the resultant protein showed oriented membrane targeting (Lazar et al., 2011), the detection of SHG signal remained elusive.

In this paper, we report our successful effort in engineering a genetically-encoded SHG chromophore. Our approach is inspired by the mechanism by which Kirsten Ras4B (K-Ras4B) interacts with plasma membranes (Welman et al., 2000). K-Ras4B, a member of the four Ras homologs which are expressed ubiquitously (H-Ras, N-Ras, K-Ras4A, and K-Ras4B), undergo switching between GDP-bound inactive and GTP-bound active states, and modulate cellular signaling of cell growth and differentiation. Ras proteins generally consist of a conserved N-terminal region and a C-terminal hyper-variable domain. The conserved N-terminal region is involved in the binding of GTP/GDP as well as the associations with effector proteins such as PI3 kinase, Raf kinase, Ral GDS, and AF6 (Welman et al., 2000). The C-terminal hyper variable domain is involved in the association with plasma-membrane. Unlike the other three members, the hypervariable domain of K-Ras4B contains a polybasic region in conjunction to the farnesylation target, which is conferred though an alternate mRNA splicing and provides the synergistic electrostatic and hydrophobic mechanism to associate with plasma-membranes (Hancock et al., 1990, 1991; Welman et al., 2000). Because electrostatic interactions of a protein with membrane surface electric-field occurs only at a short distance from the membrane, we explored whether the mechanism found in K-Ras4B may be utilized to engineer chromophores which are tightly oriented at the membrane-cytoplasm interface, a critical step toward building optical indicators of membrane potential.

## Materials and methods

### Molecular biology, cell culture, and protein modeling

We used a custom modified pCS2+ vector for heterologous expression experiments in mammalian cells. Site-directed mutagenesis was performed as described previously (Sawano and Miyawaki, 2000). HEK293T cells were cultured in Dulbecco's Modified Eagle Medium supplemented with 10% fetal bovine serum in a standard incubator (5% CO_2_, 37°C). Transfection was performed using Lipofectamine 2000 reagent (Thermo Fisher Scientific, MA) according to the manufacturer's protocol. The crystal structure for Venus (PDB#:1MYV; Rekas et al., 2002) was used as a template for molecular modeling. The mutant models were generated by using Swiss PDB Viewer software (Guex and Peitsch, 1997; http://www.expasy.org/spdbv/). The electrostatic surface potential maps for the mutant models were generated by the web-based software, eF-surf (http://ef-site.protein.osaka-u.ac.jp/eF-surf/top.do).

### Wide-field fluorescence microscopy

Cells were imaged at 24~36 h post-transfection on an inverted microscope (IX71, Olympus, Tokyo, Japan) equipped with a 75 W xenon lamp and a CMOS camera (Orca-Flash2.8, Hamamatsu Photonics, Hamamatsu, Japan). Excitation, dichroic, and emission filters used were ex500/24, dm520, and em542/27 (Semrock, N.Y.), respectively. A rotatable polarizer in the excitation light path (Sigma-Koki, Tokyo, Japan) was used to study polarization angle dependency. A 40× objective lens with a moderately low numerical aperture (UPlanFLN 40x, NA 0.75, Olympus, Tokyo, Japan) was used to minimize aperture effect. Intrinsic angle-dependency of the imaging system (g-factor) was calibrated using isotropic solution of fluorescein deposited between two coverslips.

### Second harmonic and two-photon excited fluorescence microscopy

We used two setups of laser scanning microscope. One (Columbia University) was a custom-made two-photon laser scanning microscope based on the Olympus FV-300 system (FV-300 side-mounted to a BX50WI microscope with a 60X, N.A. 1.1water immersion objective, LUMFLN 60XW) and a Ti:sapphire laser (Chameleon Ultra, Coherent). Details for the instrumentations have been described elsewhere (Nikolenko et al., 2003). Fluorescence was detected with a top-mounted Hamamatsu H7422-P40 PMT connected to a Hamamatsu C7319 preamplifier whose output was connected to a Fluoview system (Olympus). SHG was detected with a bottom-mounted similar PMT. The polarization of fundamental light was modified by a quartz zero-order half-wave plate (Newport Corporation). The other (RIKEN) is a multi-photon excitation imaging system (FV1000MPE, Olympus, Tokyo, Japan) at RIKEN BSI-Olympus Collaboration Center, equipped with a Ti-sapphire pulsed laser (InSight, Spectra-Physics, CA), an upright microscope, and a water immersion objective (LUMPlanFL/IR 60XW, N.A. 0.9, Olympus, Tokyo, Japan). Second harmonics emitted to the forward direction was collected by a condenser lens (N.A. 0.9) and detected with a photomultiplier module (Hamamatsu Photonics, Hamamatsu, Japan) in the transmission light path through a narrow band-pass filter of appropriate band. Two-photon excited fluorescence was detected using a detector in the reflection light path.

## Results

### Designing a fluorescence protein with an electrostatic surface patch

**Figure 1:**
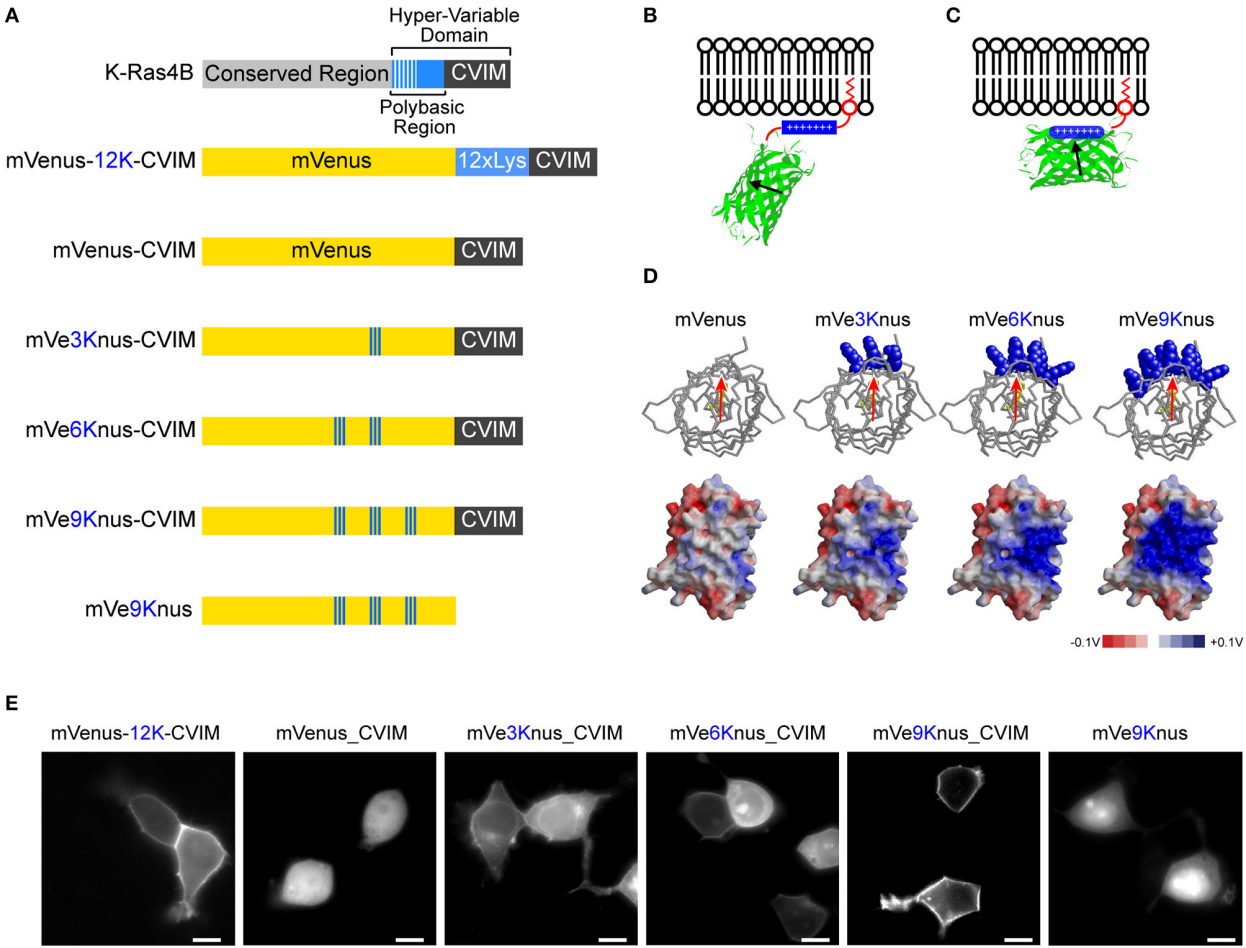
**Engineering of mVe9Knus-CVIM. (A)** Primary structures of K-ras4B as well as the six mVenus-based constructs studied. “CVIM” in the gray box represents the farnesylation signal. The engineered lysine resides in mVenus-based constructs are highlighted in blue. **(B,C)** The design of these constructs consists in transferring the polybasic region to the surface of mVenus, aiming to orient it in a geometrically defined manner at the membrane-cytoplasm interface. **(D) *Top:*** Top views of mVenus and the three mutant models. The introduced lysine residues are highlighted in the blue, space-filled models. The red arrow approximates the transition dipole moment. ***Bottom:*** Side views of electrostatic surface potential maps in these models. **(E)** Representative images of HEK293 cells expressing the corresponding constructs. Bars = 10 μm.

The hypervariable domain of K-Ras4B consists of the polybasic region followed by the tetra- peptides, Cys-Val-Ile-Met (“CVIM” in a single-letter code), which acts as the farnesylation signal (Figure 1A). It has been shown that this synergistic membrane localizing mechanism can be applicable for other host proteins such as a fluorescence protein (Welman et al., 2000). In this study, we were first interested in exploring whether the polybasic region is separable from the C-terminal farnesylation target, and transferred onto the host protein surface, constituting an electrostatic patch, so that the host is oriented to the membrane in a geometrically defied manner (Figures 1B–D).

A previous study has revealed that the amount of basic amino acids in the polybasic region, rather than the specific sequence, is essential for efficient membrane localization (Welman et al., 2000). Our experiments also confirmed this conclusion. An artificial hypervariable domain consisting of 12 lysines followed by CVIM was fused after mVenus (i.e., a yellow fluorescence protein carrying A206K mutation; Nagai et al., 2002; Zacharias et al., 2002). We found that this chimeric protein was efficiently targeted to plasma membrane in HEK293T cells (mVenus-12K-CVIM; Figures 1A,E). The removal of the polybasic region (i.e., 12 × Lys) resulted in totally cytosolic expression (mVenus-CVIM; Figures 1A,E). These observations are thus in consistent with the previous study (Welman et al., 2000) and verified the functional significance of the positive charges in the hypervariable domain of K-Ras4B.

Using mVenus-CVIM as a template, we then mutated to lysine the amino acids that build the surface of the β-barrel in order to introduce a positively charged, electrostatic patch. It has been postulated that the transition dipole moment of the GFP chromophore subtends slight angles with the vector that joins the phenolic and imidazolinone oxygen atoms of the chromophore (Rosell and Boxer, 2003; Shi et al., 2007). Aiming to direct this vector roughly perpendicular to the membrane, we generated three variants which carried three (Y200K, S202K, Q204K), six (Y200K, S202K, Q204K, S147K, N149K, Y151K), and nine (Y200K, S202K, Q204K, S147K, N149K, Y151K, L221K, F223K, T225K) substations and named them as mVe3Knus-CVIM, mVe6Knus-CVIM, and mVe9Knus-CVIM, respectively (Figures 1A,D). These residues were determined basing on the crystal structure of Venus (PDB# 1MYV). In particular, the first three amino acids (Y200, S202, and Q204) are located on the opposite side of Tyr^203^ whose phenolic group interacts with the chromophore though π-stacking. The effects of the substitutions on membrane localization were tested by expressing in HEK293 cells (Figure 1E). Membrane localizations were only partially recovered in mVe3Knus-CVIM and mVe6Knus-CVIM, but was totally recovered in mVe9Knus-CVIM (Figure 1E), demonstrating significance of the area size of electrostatic patch (Figure 1D). Finally, the effect of removing the farnesylation target from mVe9Knus-CVIM was examined in order to discriminate whether its membrane localization relies on the synergistic mechanism as before, or is just as a result of the enlarged electrostatic patch. The removed construct, mVe9Knus, did not show membrane localizations but rather showed partial nuclear localization (Figure 1E), which supported the former view.

### Polarization dependence of mVe9Knus-CVIM fluorescence

**Figure 2:**
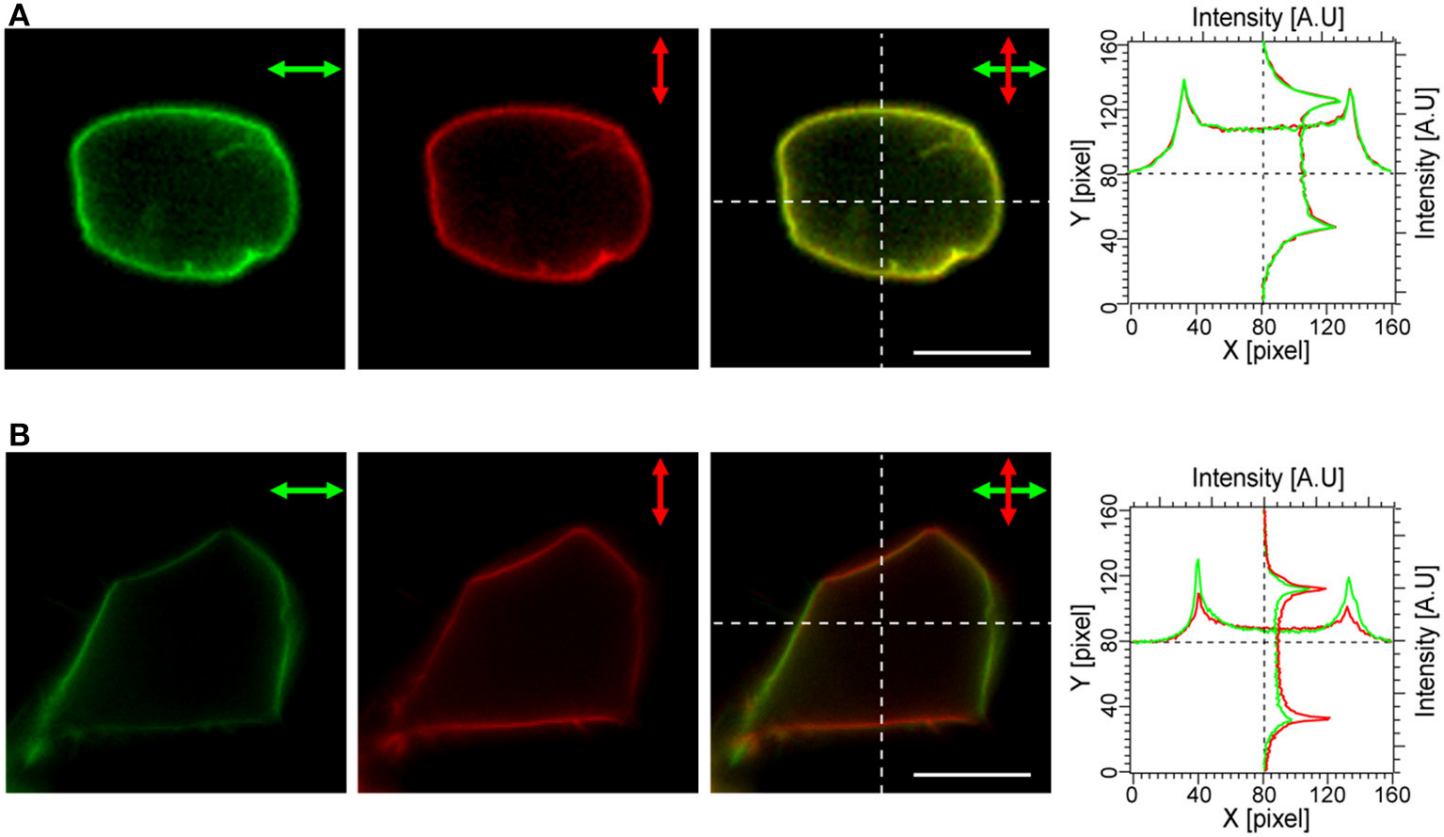
**Polarization dependency of mVe9Knus-CVIM Fluorescence**. The panels show cellular fluorescence upon excitation with horizontally (green) and vertically (red) polarized light, the merged images, as well as intensity profiles along the dotted lines for the two constructs: mVenus-12K-CVIM **(A)** and mVe9Knus-CVIM **(B)**. Bars = 10 μm.

Next, the polarization dependency of cellular fluorescence was analyzed in order to evaluate an effect of the electrostatic patch on the transition dipole orientations with respect to the membrane normal (Figure 2). The fluorescence profiles in the two images, one acquired with horizontally-polarized and the other with vertically-polarized excitation light reflected a substantial flexibility in the orientations of the transition dipole moment and were almost identical for a cell expressing mVenus-12K-CVIM, (Figure 2A). In contrast, in cells expressing mVe9Knus-CVIM, fluorescence was critically dependent on the polarization angle (Figure 2B). The merged image as well as the profile (Figure 2B) showed that mVe9Knus-CVIM at the membrane was more efficiently excited when the polarization angle was close to the membrane normal; indicating that the transition dipoles were not randomly distributed but rather oriented parallel to the membrane normal, on average. Such observed geometrical constrain was consistent with our protein design of electrostatic surface patch (Figure 1D).

### Second harmonic generation by mVe9Knus-CVIM

**Figure 3:**
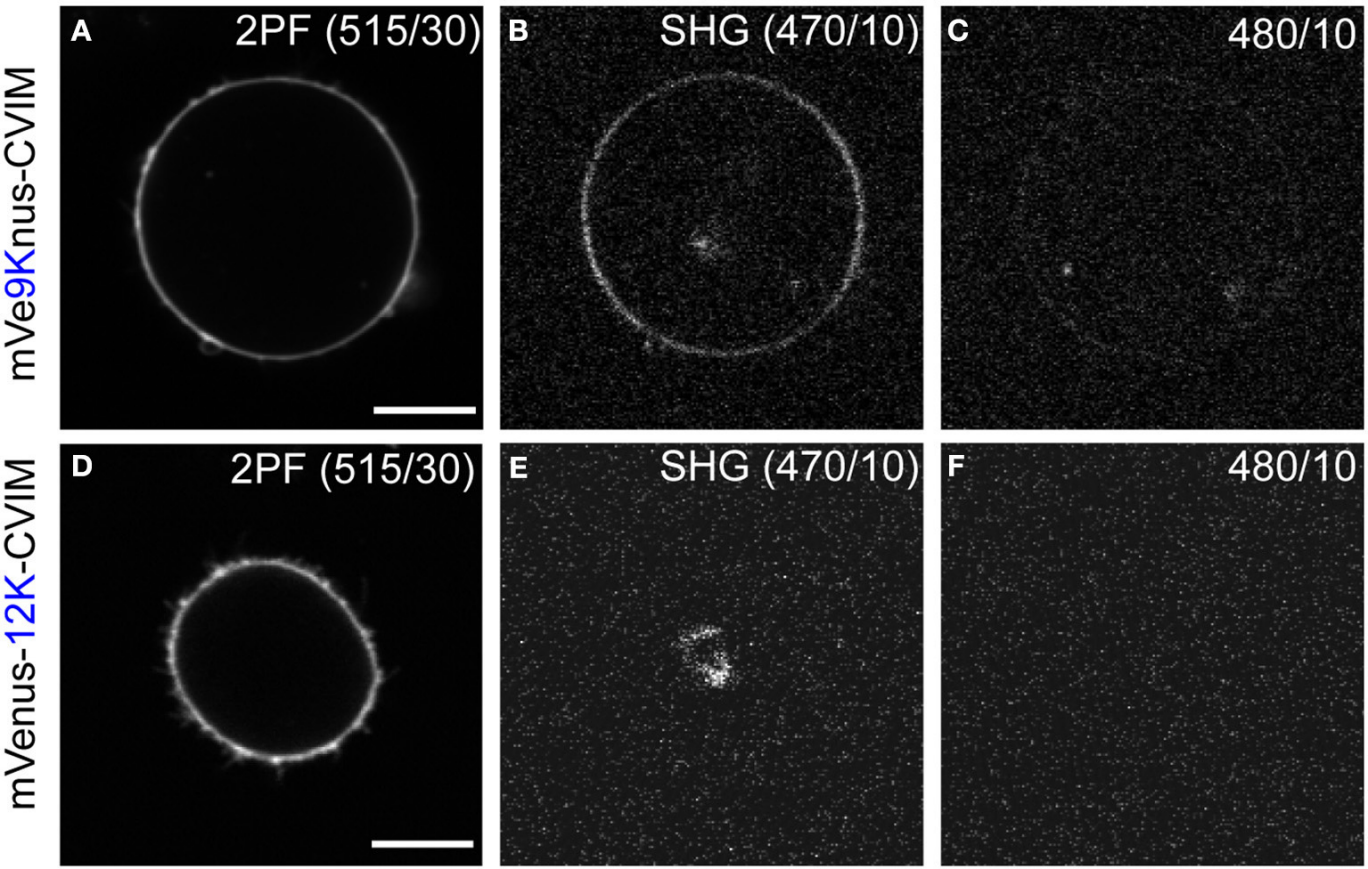
**Second harmonic imaging of mVe9Knus-CVIM. (A–C)** Images showing two-photon excited fluorescence (2PF), SHG, and off-band signals from a cell expressing mVe9Knus-CVIM, respectively. **(D–F)** Same for a cell expressing mVenus-12K-CVIM. Fundamental wavelength was 940 nm. **(B,C,E,F)** Used the same brightness/contrast settings to allow direct compa-risons. Actual optical filters used were indicated in the images. Bars = 10 μm.

**Figure 4:**
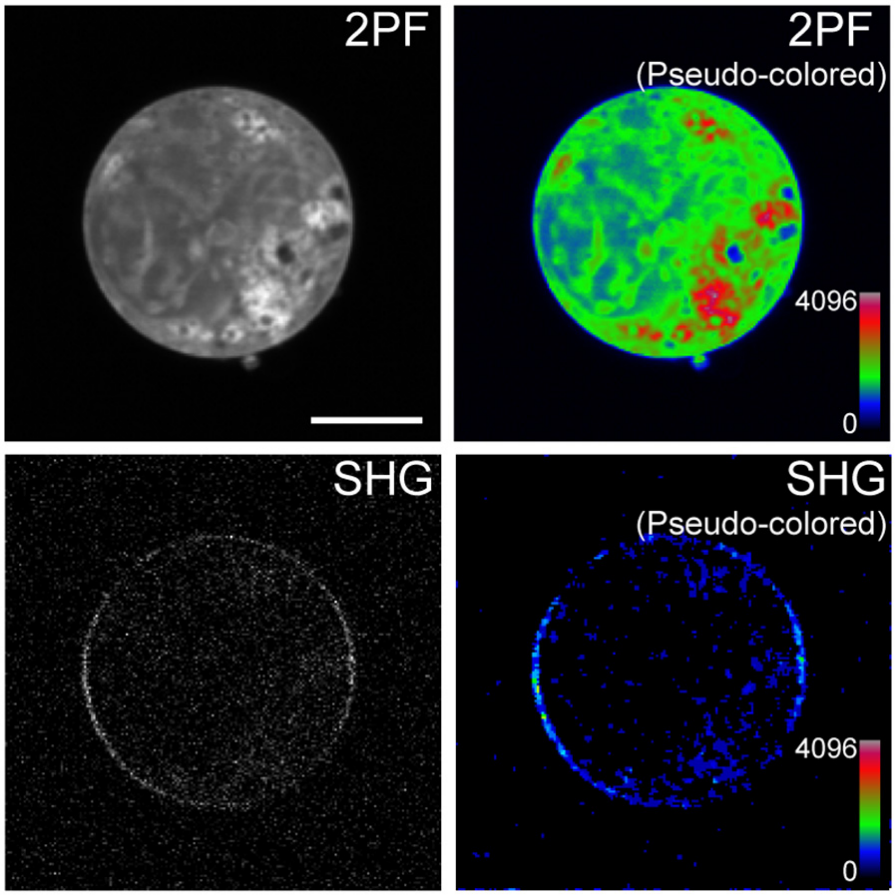
**Sensitivity of mVe9Knus-CVIM SHG to inversion symmetry**. Images show two-photon excited fluorescence (2PF) and SHG signals from an overexpressing cell in gray and pseudocolor. Proteins are more densely distributed inside the cell than at the membrane. Despeckle filter was applied prior to generate the pseudocolored SHG image (lower right panel). Bar = 10 μm.

**Figure 5:**
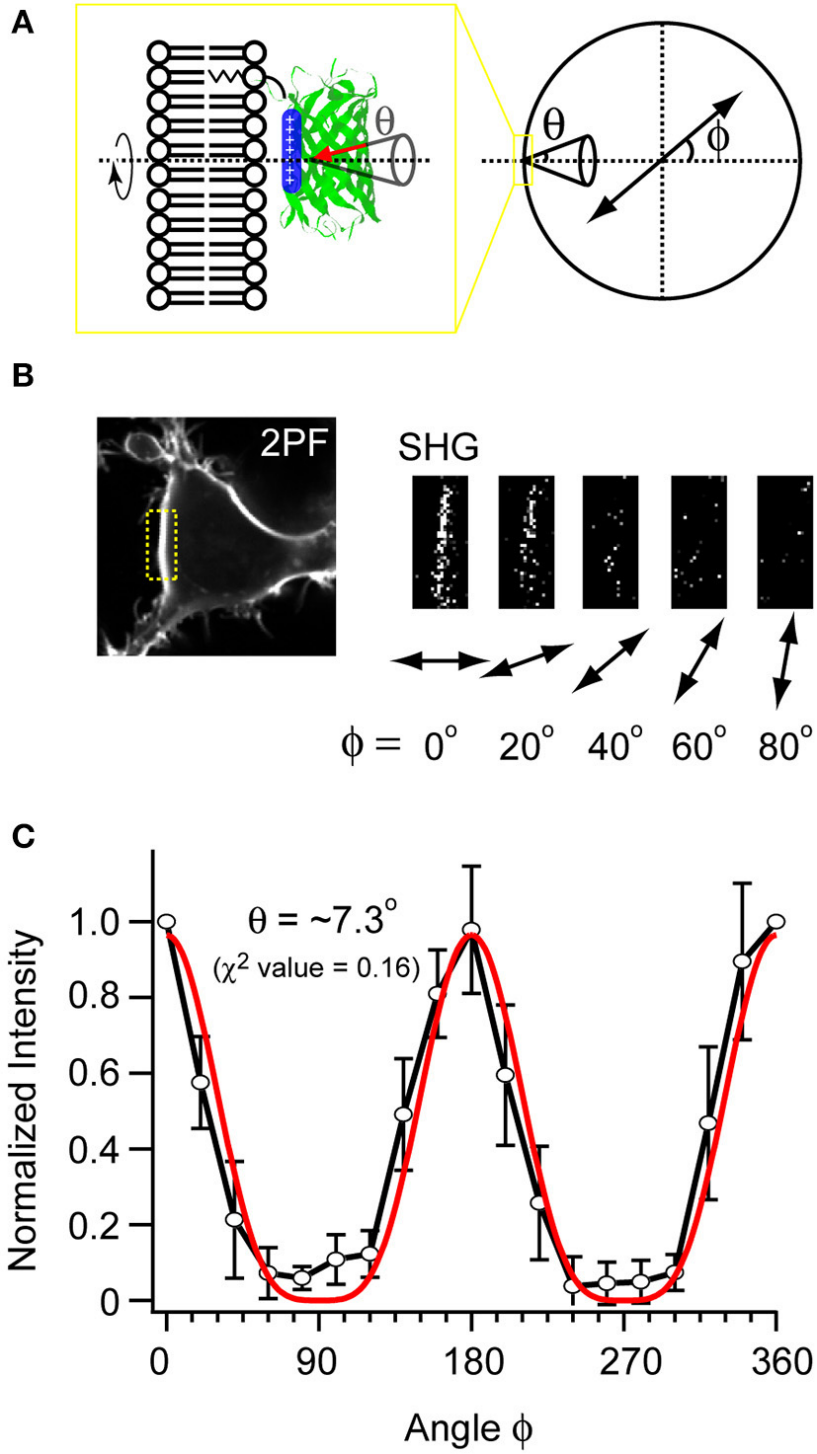
**Measuring the orientation of mVe9Knus-CVIM at the membrane interface. (A)** A scheme illustrating the parameters. ϕ: angle of the polarization plane with respect to the membrane normal. θ: tilt angle of chromophore's uniaxial hyperpolarizability with respect to the membrane normal. The cone represents the distributions on ensemble. **(B)** Example images obtained with five different polarization angles (ϕ). **(C)** The pooled data (black plot; average ± SD; *n* = 6), and its fit (red plot).

Cells expressing mVe9Knus-CVIM were then subject to SHIM. Laser wavelengths from 780 to 1064 nm were examined. The SHG band (exactly half of the fundamental wavelength) and the two-photon excited fluorescence band (480~550 nm) were separated by narrow band-pass filters. This required the fundamental wavelength to be shorter than 960 nm. SHG was extremely weak or undetectable at wavelengths below 900 nm, suggesting a resonant enhancement by electronic excitation. Under such constrains, the optimal wavelengths to obtain SHG signals were 940~950 nm. Typically, a field of view of 70 × 70 μm was scanned as a 320 × 320 pixel image at a pixel dwell time of 40 μs. Laser power as large as 30~40 mW at the sample was normally needed to detect clear second harmonic signals. Representative two-photon fluorescence and SHG image upon illumination at 940 nm are shown (Figures 3A,B). The possibility that the signal through a 470/10 nm band-pass filter (Figure 3B) originates from blue-edge of the two-photon excited fluorescence was excluded because the replacement by a 480/10 nm filter practically eliminated the signal (Figure 3C). SHG signals were not detected from membrane of the cells expressing mVenus-12K-CVIM (Figures 3D–E), reflecting the critical importance of the anisotropic orientation control of the chromophores. Weak endogenous SHG signals were occasionally detected from the center of the cells where no fluorescence was observed (Figures 3B,E). They could reflect SHG from endogenous structural proteins such as microtubules (Campagnola et al., 2002). In addition, the sensitivity to local centrosymmetry, the nature of SHG signal, was confirmed in the cells overexpressing mVe9Knus-CVIM (Figure 4). In these cases, although most of the protein was concentrated inside the cell rather than at the membrane-cytoplasm interface, as revealed by the pseudo-colored fluorescence image, second harmonic signals only arise from the interface, where the inversion symmetry is broken. Finally, we quantitatively addressed the orientation of mVe9Knus-CVIM at the membrane-cytoplasm interface. Under the simplifying assumption that the hyperpolarizability tensor of the chromophore is dominated by an element along its long molecular axis, the dependency of SHG from membrane-bound dyes on the polarization angle can be analytically expressed as Jiang et al. (2007):

(1) $$\begin{array}{l} SHG\propto \{{\left[<\cos^{3}\theta >\cos^{2}\phi +<\cos\theta \sin^{2}\theta >\sin^{2}\phi /2\right]}^{2}\\ \;+<\cos\theta \sin^{2}\theta {>}^{2}\sin^{2}\phi \cos^{2}\phi \}, \end{array}$$

where θ and ϕ are the angles of membrane normal subtended from the dye axis, and that from the polarization plane of fundamental light, respectively (Figure 5A). The angle brackets denote ensemble average. Figures 5B,C show representative and pooled data, respectively. When a fixed θ value was assumed for the ensemble, the curve fitting resulted in the tilt angle θ of ~7.3°.

## Discussion

In this study we have successfully engineered a GFP-based SHG membrane chromophore, even though one may not in principle expect strong SHG from GFP-based membrane proteins, because the SHG intensity from a chromophore is proportional to the square of its density (Shen, 1989) and the maximum dipole density is intrinsically limited by the size of β-barrel (Roorda et al., 2004). These difficulties were likely compensated by a highly-ordered protein orientation at the membrane-cytoplasm interface. For mVe9Knus-CVIM, the tile angle (θ = ~7.3°) was smaller than that measured for the styryl dye FM4-64 (θ = ~36°) (Jiang et al., 2007), although the insertion mechanisms of FM4-64 and mVe9Knus-CVIM are very different (lipophilic attachment for FM4-64 vs. electrostatic for mVe9Knus-CVIM).

FM4-64 and other organic dyes bound in cell membrane produce robust voltage dependent second harmonics (Campagnola et al., 1999; Millard et al., 2003b, 2004; Nemet et al., 2004), and have been successfully applied to monitor electrical activity in fine membrane structures (Nuriya et al., 2006). Various effects, from direct to rather indirect ones, could serve as the mechanisms for voltage sensitivity, including electro optic, or chromic effect, translocation, reorientation, and redistribution (Moreaux et al., 2003; Pons et al., 2003; Jiang et al., 2007). It would be interesting to ascertain whether the second harmonics from mVe9Knus-CVIM is sensitive to transmembrane voltage. Because the Debye length in physiological ionic condition is probably smaller than 10 Å, the chromophore may lie outside of the substantial surface field. At the same time, it is possible that, like it has been estimated for nanoparticles (Dr. J. Owen, personal communication), the electric field could protrude from the membrane and engulf a molecule that excludes ions. Indirect effects cannot be ruled out also, since our protein is actually oriented to the membrane through the electrostatic interaction with the short-distance field. In principle, the degree of interaction and orientation could be subject to modulation. Assuming membrane capacitance C of ~1 μF/cm^2^, a voltage change ΔV of ~100 mV will associate change in the surface charge:

(2) $$\Delta Q=C\Delta V\approx 10^{-7}[C/cm^{2}]\approx 1/15000[e/{Å}^{2}],$$

which is equivalent to ~1% of net cytoplasmic surface charge of typical phospholipid composition (1/100~1/200 [e/Å^2^]; Smith et al., 1993; Xu and Loew, 2003). In preliminary experiments, we performed pilot experiments of simultaneous patch-clamp and SHG measurements, but have not detected clear voltage dependent signals so far. Nevertheless, we consider that a solid conclusion regarding this issue has not been obtained since poor signal-to-noise ratio, which has hampered us to perform precise measurements. Use of higher laser power may not be appropriate since our second harmonic signal most likely relies on resonant enhancement through electronic excitation. Notably, SHG is proportional to square of the dipole density, and thus our signal is highly sensitive to chromophore destruction. In spite of this, we envision two future directions toward improvements. The first is to exploit the diversity of GFP-like proteins, which could differ widely not only in chromatic properties, but also in non-linear properties. In fact, the protonated form of Dronpa, a photo-switchable coral fluorescence protein (Ando et al., 2004), has been shown to exhibit large high non-linear hyperpolarizability (Asselberghs et al., 2008). At this point, many other GFP-like proteins still remain unevaluated for SHG. Thus, performing a proper survey with subsequent protein engineering as in the present study could lead to a better performing SHG chromophores. The second direction for future improvement is more precise control of the electrostatic property of the β-barrel surface in terms of the area size and also the position. As observed in the constructs with less surface lysine (Figures 1D,E), the protein order at the interface alters the balance between the electrostatic energy stabilization and the thermal relaxation. By considering derivate of the Equation (1), it is anticipated that the response to the electrostatic perturbation will depend on basal tile angle, θ. Thus, there might be an optimal surface electrostatic condition where ΔQ would most effectively influence the signal.

In addition, in terms of mechanisms of protein-membrane association, there are some other proteins (e.g., Src, MARCKS, HIV-1 gag) which are known to rely on the electrostatic mechanism similar to K-Ras4B. While all these proteins likewise possess apparent basic clusters in their primary structures, the present study clearly demonstrated that the basic amino acids are not necessarily clustered in the primary structure whenever they constitute a basic patch in the tertiary structure. Thus, might be more natural proteins which have not yet been recognized to interact electrostatically.

Compared to the recent achievements building genetically-encoded probes basing on fluorescence (Miyawaki, 2011), the design of protein probes based on SHG has remained behind. In this study, as a first step, we show that genetically-encoded chromophores can be in fact anisotropically arranged beneath the membrane through the artificial control of the electrostatic properties of the protein surface. Although still faint, the second harmonic signals from mVe9Knus-CVIM could be improved for wider biological use. Using this strategy for orientation control of membrane chromophores could enable further development of genetically-encoded probes for membrane potential imaging.

### Conflict of interest statement

The authors declare that the research was conducted in the absence of any commercial or financial relationships that could be construed as a potential conflict of interest.
